# A genetic algorithm–optimized EWMA control chart integrating machine learning-based risk prediction for multistage thyroid cancer treatment monitoring

**DOI:** 10.1371/journal.pone.0347876

**Published:** 2026-10-06

**Authors:** Mohammad Rasouli, Elham Rahimi, Arshia Ilaty

**Affiliations:** 1 Caspian Faculty of Engineering, College of Engineering, University of Tehran, Tehran, Iran; 2 Donald Bren School of Information and Computer Sciences, University of California Irvine, Irvine, California, United States of America; University of Hamburg: Universitat Hamburg, GERMANY

## Abstract

Effective monitoring of thyroid cancer treatment requires timely detection of clinically meaningful changes across complex, multistage care pathways. Traditional monitoring approaches often rely on static thresholds and periodic assessments, limiting their sensitivity to subtle yet important process deviations and potentially delaying clinical intervention. This study aimed to develop and evaluate an integrated, data-driven framework that combines deep learning–based risk prediction with genetic algorithm–optimized statistical process monitoring to enhance thyroid cancer treatment surveillance. Informative clinical variables were first identified using the Information Functions algorithm, and a Multilayer Perceptron (MLP) neural network was then employed to predict patient-specific treatment risk, capturing nonlinear relationships within longitudinal electronic medical record data. The resulting risk estimates were sequentially incorporated into Exponentially Weighted Moving Average (EWMA) control charts to enable continuous process monitoring. To further improve chart performance, a genetic algorithm was used to optimize EWMA parameters, enhancing accuracy and sensitivity while maintaining chart stability. The results demonstrate that the MLP model outperforms conventional machine learning approaches in risk prediction across multiple evaluation metrics. Genetic algorithm–optimized EWMA charts exhibit smoother trajectories, improved stability, and more reliable detection of clinically meaningful deviations compared with non-optimized charts. The consistency of these improvements across multiple features suggests that GA-based optimization enhances overall control chart performance rather than feature-specific behavior. This hybrid framework demonstrates the feasibility of integrating machine learning–derived risk prediction with optimized statistical process control for longitudinal treatment monitoring. The proposed approach supports proactive treatment surveillance, risk-stratified follow-up, timely clinical intervention, and data-driven decision-making in thyroid cancer care. Furthermore, the framework has the potential to enable continuous and adaptive monitoring across complex multi-stage treatment pathways if integrated with electronic medical record (EMR) systems in future implementations.

## 1. Introduction

Thyroid cancer ranks seventh among all malignancies worldwide and exhibits a more rapidly increasing incidence rate than any other cancer type, accompanied by a slowly rising mortality rate [1,2]. Once considered a relatively rare malignancy, thyroid cancer has become the most common cancer of the endocrine system [3], and its treatment presents significant clinical challenges due to disease heterogeneity and variability in patient response [4–6]. The treatment process often involves repeated imaging, fine-needle aspiration biopsy, thyroidectomy, radioactive iodine therapy, and lifelong thyroid hormone replacement therapy [3]. In the current clinical workflow, monitoring treatment progress, predicting patient outcomes, and adjusting treatment plans rely heavily on clinical indicators such as thyroglobulin levels, tumor size, and administered radioactive iodine dose [3]. However, routine clinical decision-making based on periodic assessments and static thresholds may fail to capture early or subtle changes in disease status, resulting in delayed interventions, increased exposure of low-risk patients to invasive procedures and radiation, and substantial financial burden on patients and healthcare systems [3]. Moreover, the growing volume and complexity of clinical data may limit clinicians’ ability to fully leverage available information [7], further increasing the risk of suboptimal outcomes [8]. These challenges highlight the need for more precise, data-driven treatment process monitoring and risk stratification approaches that support timely decision-making while balancing the risks of overtreatment and undertreatment, with the goal of reducing morbidity and mortality while avoiding unnecessary interventions in low-risk patients.

Statistical process control (SPC) methods have been increasingly explored for monitoring healthcare and treatment processes due to their ability to detect deviations in system behavior over time [9–12]. In particular, Exponentially Weighted Moving Average (EWMA) control charts, designed to assign greater weight to recent observations, have been applied in healthcare settings to monitor surgical outcomes and multistage treatment processes, demonstrating improved sensitivity to small and gradual shifts compared with traditional control charts [13,14]. Rasouli et al. introduced a risk-based framework using multivariate EWMA group control charts to monitor multistage treatment processes, highlighting the potential of SPC techniques for healthcare process monitoring [15–17]. In parallel, machine learning and deep learning models have been widely adopted in healthcare for risk prediction and outcome modeling, leveraging large-scale clinical data to capture complex and nonlinear relationships. Consequently, statistical and machine learning approaches have increasingly been incorporated into healthcare process modeling to better represent system dynamics and patient heterogeneity. Yeganeh et al. proposed a machine learning–based framework for monitoring multistage healthcare processes, demonstrating the value of predictive modeling in guiding process-level decision-making [18,19]. More recently, risk-adjusted EWMA control charts have been extended using statistical learning and machine learning models to further enhance monitoring performance in clinical applications. Noor-ul-Amin et al. proposed a risk-adjusted EWMA control chart based on support vector machines for monitoring cardiac surgery outcomes [14], while Abbas et al. developed a machine learning–based adaptive EWMA framework to dynamically improve control chart performance [20]. Ahmadini et al. further advanced this line of research by integrating artificial neural networks into risk-adjusted EWMA control charts, demonstrating enhanced sensitivity and robustness in healthcare monitoring scenarios [21].

Despite these advances, existing statistical methodologies often rely on linear risk-adjustment or static control chart parameters, which may fail to capture the high-dimensional, non-linear dynamics inherent in longitudinal patient data. While hybrid SPC-ML models have shown promise, current frameworks frequently overlook the optimization of control chart parameters, potentially leading to increased false-alarm rates or delayed detection of clinical shifts.

The performance of EWMA control charts is highly sensitive to the choice of the smoothing parameter (λ) and the control-limit width (L), which are conventionally fixed or selected from tabulated average run length (ARL) values derived under the assumptions of known process parameters and normally distributed, independent observations [22]. These assumptions are difficult to justify when the monitored quantity is a model-derived risk score with an unknown and potentially non-normal distribution. Furthermore, when in-control parameters must be estimated rather than assumed to be known, EWMA run-length performance can deteriorate, increasing false alarms while reducing sensitivity [23]. Manual or grid-based tuning also becomes impractical when monitoring is extended across multiple features and treatment stages, as the parameter space expands. Therefore, a single automated and reproducible tuning procedure is preferable to manual, per-chart parameter selection.

Consequently, there is a lack of unified frameworks that integrate dynamic deep learning–based risk prediction with automated optimization for monitoring multistage treatment pathways. This is particularly critical in thyroid cancer care, where patient heterogeneity and complex trajectories demand adaptive surveillance tools capable of detecting subtle deviations to prevent adverse clinical outcomes.

This gap motivates the development of an integrated framework that combines machine learning–driven risk estimation with genetic algorithm–optimized EWMA control charts, enabling more sensitive, adaptive, and clinically meaningful monitoring of treatment processes. Informative clinical variables are first identified using the Information Functions algorithm, which enhances the accuracy and efficiency of the risk prediction model by ensuring that only the most relevant data are used in predictive modeling. Risk prediction is then performed using a Multilayer Perceptron (MLP) model capable of capturing complex, nonlinear relationships in treatment-related data. The resulting risk estimates are subsequently embedded within EWMA control charts to enable continuous and dynamic monitoring of treatment trajectories. As these risk-score statistics have an unknown, potentially non-normal distribution and are monitored across multiple features and treatment stages, a distribution-free, derivative-free optimization strategy is preferable to fixed or tabulated parameter selection. Genetic algorithms, as a population-based, derivative-free approach, have been applied successfully to the optimal design of univariate and multivariate EWMA charts [24]. We therefore employ a genetic algorithm to optimize the EWMA parameters, providing a principled and extensible basis for producing more conservative charts that maintain chart stability while reducing unnecessary alarms. The rationale for this approach is clinical rather than purely statistical. In longitudinal patient surveillance, an overly sensitive chart generates frequent non-actionable alarms, which can exceed 70% of all alarms and contribute to alarm fatigue and clinician desensitization [25,26], as well as to unnecessary clinical follow-up and intervention which, in thyroid cancer, are associated with avoidable complications and considerable financial burden [27]. By optimizing the control-chart parameters accordingly, the framework prioritizes specificity and lowers the burden of unnecessary alarms and follow-up.

Collectively, this framework advances precision monitoring in thyroid cancer treatment by bridging predictive modeling and adaptive statistical process control, enabling earlier detection of treatment deviations, supporting timely clinical intervention, and improving patient-centered decision-making.

## 2. Methods

### 2.1. Methodological framework

The proposed model framework consists of four main stages:

1. **Data preprocessing:** The dataset from patients with thyroid cancer was preprocessed to ensure consistency and standardization. This step included data cleaning, handling missing values, and normalization of variables.
2. **Risk prediction modeling:** Relevant features of the preprocessed data were selected using the Information Functions algorithm to ensure that only informative and high-importance variables were included in the modeling process. A MLP neural network was then designed and implemented as a risk prediction model for thyroid cancer patients.
3. **Control chart design and implementation:** The prediction outputs produced by the MLP model were used as inputs for constructing control charts. These charts were designed to monitor the treatment process over time and detect significant deviations. For this purpose, an EWMA control chart was employed to identify shifts and trends in the treatment process.
4. **Genetic algorithm–based optimization of the EWMA control chart:** The EWMA control chart parameters, including the smoothing parameter (λ) and control limits (L), were optimized using a genetic algorithm (GA). The GA simulates the process of natural selection to identify optimal parameter values that enhance the performance of the control chart.

Overall, this integrated framework combines deep learning–based predictive modeling with genetic algorithm–optimized statistical process monitoring, providing a comprehensive and efficient approach to improving treatment monitoring and patient outcomes. Fig 1 illustrates the overall structure of the proposed methodology.

**Fig 1:**
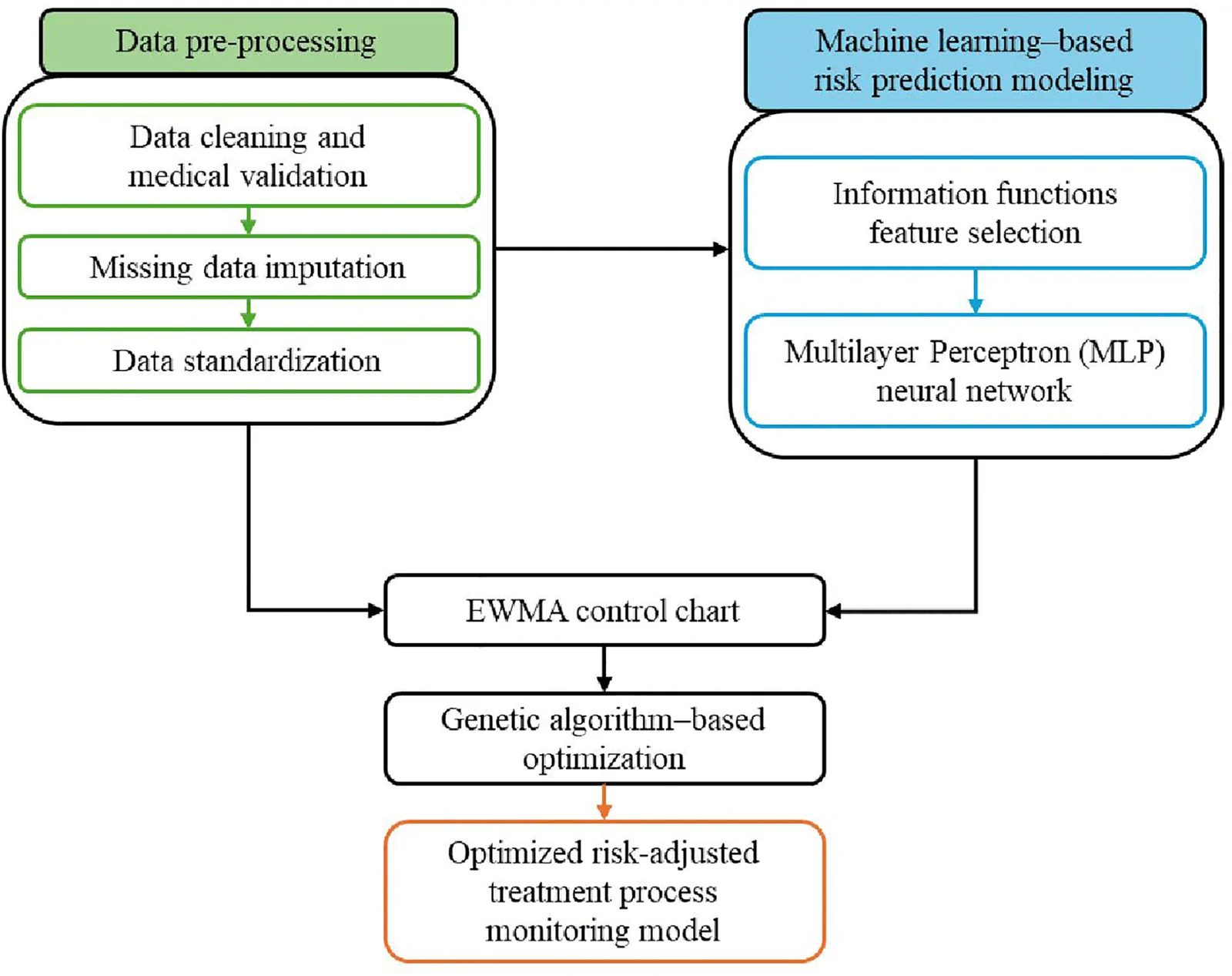
Methodological framework.

### 2.2. Dataset description

In this study, secondary data analysis was conducted using data extracted by Rasouli et al. [17] from the electronic medical records (EMRs) of a hospital located in Tehran, Iran, between 2009 and 2013. The dataset consists of 80 patients who underwent two-stage thyroid cancer surgery and includes de-identified demographic information, medical history, test results, surgical reports, and treatment stages. All extracted medical record data were verified for completeness and accuracy by medical experts. In addition, lifestyle habits, such as smoking status and alcohol consumption, collected through questionnaires and patient interviews, were extracted from the electronic medical records.

#### 2.2.1. Ethics statement.

This study involved secondary analysis of de-identified electronic medical record data from Shariati Hospital, Tehran, Iran. The dataset was fully de-identified prior to access, and no direct or indirect patient identifiers were available to the research team. No new data collection or patient contact was performed. In accordance with the regulations of the Shariati Hospital Ethics Committee, secondary use of de-identified data from the hospital’s data center is exempt from ethics-committee review when conducted under the institution’s data use agreement; the present study was carried out entirely under that agreement and therefore qualified for this exemption. The data use agreement authorized only designated members of the approved research team to access the de-identified data, and solely through the hospital’s physical server, without any capability to transfer or download the underlying data; only analytical results may be extracted and published. Informed consent was waived given the retrospective, fully de-identified nature of the analysis.

### 2.3. Data preprocessing

A multi-step data preprocessing pipeline was implemented to ensure data quality and consistency. First, data cleaning was performed. Records with incorrect values, either due to entry errors or discrepancies, were identified and corrected by medical experts. If a correction was deemed invalid by medical expert opinion, the data were removed. Missing values were identified and, when possible, imputed using the mean of the corresponding feature. Next, feature normalization was applied using the Min–Max scaling method to maintain consistent scales across all variables, reducing scale variations and preventing any feature from disproportionately influencing the modeling process. Finally, standardization was performed using the Z-score method so that each feature had a mean of zero and a standard deviation of one, ensuring uniform scaling across the dataset and improving the performance of the machine learning models.

### 2.4. Risk prediction modeling

In this stage, we applied feature selection and advanced machine learning techniques to enhance the accuracy and efficiency of risk prediction models for thyroid cancer patients. The Information Functions algorithm was used to identify the most informative variables, which were then fed into a MLP model. Model performance was evaluated using multiple metrics, including accuracy, 5-fold cross-validation accuracy, area under the receiver operating characteristic curve (AUC), and F1 score. The predictive performance of several additional machine learning models, Linear Regression, Random Forest, Support Vector Machine (SVM), K-Nearest Neighbors (KNN), and Deep Neural Network (DNN), was also assessed and compared with that of the MLP, facilitating the selection of the model with optimal predictive performance.

#### 2.4.1. Feature selection based on information functions.

After data preprocessing, the Information Functions algorithm was employed to identify the features with the greatest impact on prediction. Feature selection using information functions is a robust and effective approach for identifying the most relevant variables associated with the target outcome in a dataset. This method leverages concepts from information theory, including entropy and mutual information, to quantify the dependency and relationships between each feature and the target variable. In this study, the effect of MI-based feature selection on treatment-process monitoring is realized through the risk-prediction model. By retaining the most informative features, the selection improves the quality of the predicted risk scores, which are the quantities monitored by the EWMA control charts. As these scores drive the monitoring stage, any improvement in predictive performance is reflected in the resulting control-chart signals. The following section describes the methodology for feature selection based on information functions, along with the corresponding mathematical equations.

Entropy is a measure of the uncertainty or unpredictability of a random variable. The entropy of a random variable X with a probability distribution P(x) is defined in Eq. (1), where H(X) is the entropy of variable X, P(x) is the probability of occurrence of value x, and the summation is over all possible values of x.


$$\begin{array}{c} \;H(X)=-\sum_{x\in X}P(x)\text{log}P(x) \end{array}$$ (1)

Mutual information is a measure of the amount of information shared between two random variables, X and Y. The mutual information between X and Y is defined in Eq. (2), where I(X;Y) represents the mutual information between X and Y, P(x,y) is the joint probability of occurrence of values x and y, and P(x) and P(y) are the probabilities of occurrence of x and y, respectively.


$$\begin{array}{c} I(X;Y)=\sum_{x\in X}\sum_{y\in Y}P(x,y);\text{log}\frac{p(x,y)}{p(x)p(y)} \end{array}$$ (2)

Mutual information quantifies the reduction in uncertainty of Y given X, and vice versa. A feature is considered important if its mutual information with the target variable is high.

#### 2.4.2. Multi-layer perceptron neural network.

The MLP neural network is one of the most widely used models in machine learning, capable of effectively handling nonlinear and complex data. This section outlines the structure of the MLP, its mathematical equations, hyperparameters, and implementation. An MLP consists of several layers: the input layer, where the number of neurons equals the number of input features; hidden layers, which may consist of one or more layers that learn the hidden features of the data; and the output layer, where the number of neurons corresponds to the number of continuous clinical features being predicted.

For each neuron in layer l, the weighted input is calculated as shown in Eq. (3), where $W^{\left(l\right)}\;$is the weight matrix for layer l, $a^{\left(l-\text{1}\right)}$ is the output of the neurons in the previous layer (layer l−1), and $b^{\left(l\right)}$ is the bias of layer l.


$$\begin{array}{c} z^{\left(l\right)}=w^{\left(l\right)}a^{\left(l-\text{1}\right)}+b^{\left(l\right)} \end{array}$$ (3)

The output of the neurons after applying the activation function is calculated as shown in Eq. (4), where σ denotes the activation function. Common activation functions include sigmoid, hyperbolic tangent, and ReLU.


$$\begin{array}{c} a^{\left(l\right)}=\sigma (z^{l}) \end{array}$$ (4)

To train the neural network, a loss function is employed to quantify the difference between the predicted and actual outputs. One commonly used loss function is the cross-entropy loss, defined as Eq. (5), where m is the number of training samples, $y_{i}$ is the actual value of the i−th sample, and ${\hat{y}}_{i}$ is the predicted value of the i−th sample.


$$\begin{array}{c} \;l=-\frac{\text{1}}{m}\sum_{i=\text{1}}^{m}[y_{i}-\text{log}{\hat{y}}_{i}+\left(\text{1}-y_{i}\right)\text{log}\left(\text{1}-{\hat{y}}_{i})\right] \end{array}$$ (5)

The weights and biases of the network are updated using the backpropagation algorithm. In this algorithm, the gradient of the loss function with respect to the weights and biases is computed, and updates are performed as shown in Eqs. (6) and (7), where η is the learning rate. Table 1 summarizes the hyperparameter values used for implementing the MLP in this study, and Fig 2 illustrates the structure of the MLP algorithm. Training proceeded until the loss converged, and stable convergence without overfitting was confirmed by the consistency of performance across the 5-fold cross-validation and the multiple evaluation metrics reported in Table 4.

**Table 1:** Hyperparameter values used for implementing the multilayer perceptron (MLP) neural network.

Hyperparameter	Value	Description
Number of Hidden Layers	1	One hidden layer with 100 neurons
Neurons per Hidden Layer	100	Number of neurons in the hidden layer
Activation Function	ReLU	ReLU activation function
Solver	Adam	Adam optimizer
Learning Rate	0.001	Initial learning rate
Number of Epochs	500	Number of epochs for training
Batch Size	Scikit-learn default	Number of samples processed per training step; default value used

**Fig 2:**
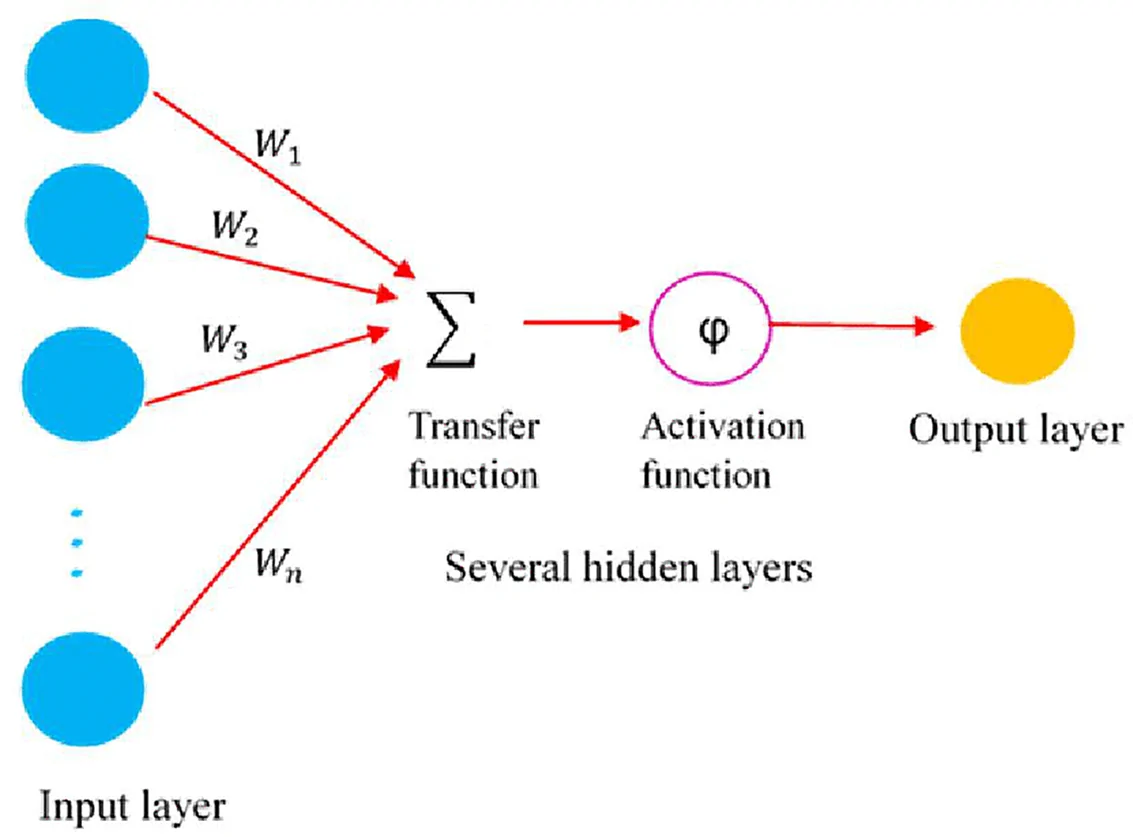
Structure of the multilayer perceptron (MLP) neural network.


$$\begin{array}{c} \;w^{\left(l\right)}=w^{\left(l\right)}-\eta \frac{\partial \text{L}}{\partial w^{\left(l\right)}} \end{array}$$ (6)

$$\begin{array}{c} \;b^{\left(l\right)}=b^{\left(l\right)}-\eta \frac{\partial \text{L}}{\partial b^{\left(l\right)}} \end{array}$$ (7)

The hyperparameter values in Table 1 correspond to established default settings of the multilayer perceptron, chosen to provide a robust, reproducible configuration suited to a dataset of limited size while minimizing overfitting risk. We retained these validated defaults and assessed generalization using 5-fold cross-validation.

### 2.5. Design and implementation of the control chart

Control charts are statistical tools used to assess whether a process is operating within a state of statistical control. In this study, an EWMA control chart was designed and implemented with the primary objectives of monitoring and evaluating the thyroid cancer treatment process and detecting significant deviations from expected performance, which may indicate potential issues requiring intervention.

The construction of the control chart follows the standard two-phase framework of statistical process control: a Phase I calibration step, in which the in-control mean (μ) and standard deviation (σ) of each feature are estimated, and a Phase II step, in which the resulting limits are applied to monitor patient trajectories. Due to the limited size of the available single-institution dataset, an independent Phase I calibration cohort was not available; the in-control parameters were therefore estimated from the study dataset and applied to monitor the same patients’ trajectories.

The following steps outline the design and implementation of the control chart:

The EWMA statistic for each variable X at time t is calculated as shown in Eq. (8), where $z_{t}$ is the EWMA at time t, $x_{t}$ is the observed value at time t, and $z_{t-\text{1}}$ is the EWMA at time t−1. The initial value, $z_{\text{0}}$, is typically set to the mean of the historical data.


$$\begin{array}{c} \;z_{t}=\lambda \;x_{t}+(\text{1}-\lambda )z_{t-\text{1}} \end{array}$$ (8)

The control limits for the EWMA chart are calculated as shown in Eqs. (9) and (10). In these equations, ${UCL}_{t}$ is the upper control limit at time t, ${LCL}_{t}$ is the lower control limit at time t, μ is the mean of the historical data, σ is the standard deviation of the historical data, and L is the control limit multiplier, typically set to 3 for 3-sigma control limits.


$$\begin{array}{c} {UCL}_{t}=\mu +L\sigma \sqrt{\frac{\lambda }{\text{2}-\lambda }(\text{1}-(\text{1}-\lambda {)}^{\text{2}t})} \end{array}$$ (9)

$$\begin{array}{c} \;{LCL}_{t}=\mu -L\sigma \sqrt{\frac{\lambda }{\text{2}-\lambda }(\text{1}-(\text{1}-\lambda {)}^{\text{2}t})} \end{array}$$ (10)

To implement the control chart, the key features of the thyroid cancer patients identified during the design phase were selected from the preprocessed data and used as inputs for the chart. Next, the mean (μ) and standard deviation (σ) for each selected variable were calculated using historical data. For each time point t, the EWMA statistic $\left(Z_{T}\right)$ for each variable was calculated. The upper control limit (${UCL}_{t}$) and lower control limit (${LCL}_{t}$) at each time point t were also calculated using Eqs. (9) and (10). Finally, the EWMA statistics, along with the control limits, were plotted to visualize process performance, and any points outside the control limits were identified as potential signals of process changes that require further investigation. Fig 3 illustrates the implementation steps of the EWMA control chart.

**Fig 3:**
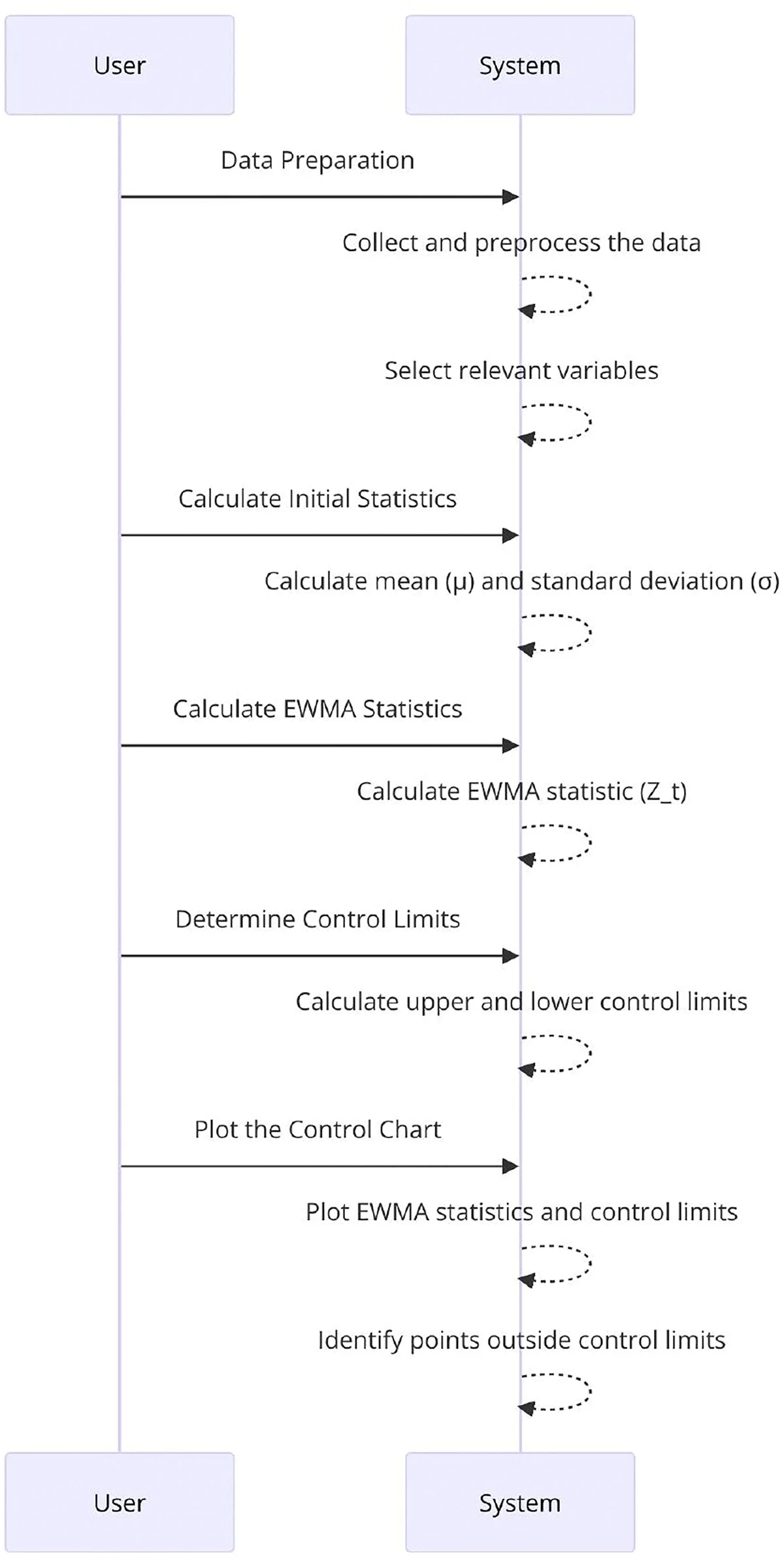
Implementation steps of the exponentially weighted moving average (EWMA) control chart.

### 2.6. Genetic algorithm–based optimization of the EWMA control chart

Although the EWMA control chart is a powerful statistical tool for monitoring process performance over time and detecting significant deviations that may indicate potential issues, optimizing its parameters can substantially enhance its sensitivity and specificity, leading to improved process control and enabling more timely interventions. Therefore, a GA was employed to optimize the parameters of the EWMA control chart, specifically the smoothing parameter (λ) and control limits (L).

Although the EWMA chart involves only two design parameters, their selection is not trivial in this setting. Conventional selection relies on tabulated values derived under the assumptions of normality and known in-control parameters; here, the monitored quantity is a model-derived risk score with an unknown, potentially non-normal distribution, and its in-control parameters must be estimated from limited data, which is known to degrade run-length performance [23]. Moreover, monitoring is performed across multiple features and treatment stages, so a single automated and reproducible tuning procedure is preferable to manual, per-chart selection. Genetic algorithms provide such a procedure and have been applied successfully to the optimal design of univariate and multivariate EWMA charts [24]; we therefore adopt a GA as a general-purpose, distribution-free optimizer within this framework.

The genetic algorithm is an optimization technique inspired by the principles of natural evolution and selection. This algorithm operates through a process that involves generating an initial population, evaluating fitness, selecting superior solutions, and inducing crossover and mutation to preserve diversity, iterating these steps until a satisfactory level of fitness is achieved.

The GA was initialized by generating a population of candidate solutions (chromosomes), each representing a set of EWMA control chart parameters, λ and L. The initial population was generated randomly within predefined bounds to ensure a diverse set of potential solutions. The fitness of each chromosome was evaluated using a fitness function that assesses control chart performance, defined as the number of observations falling outside the control limits. A lower number of out-of-control observations corresponds to a better fitness score, indicating that the selected parameters are more effective in maintaining process stability. The fittest chromosomes were selected as parents for the next generation using selection methods such as tournament selection or roulette wheel selection, ensuring that higher-performing solutions had a greater probability of passing their genes to the next generation. New offspring were generated through crossover and mutation to maintain genetic diversity. Crossover involved combining segments of two parent chromosomes to produce new offspring, while mutation introduced random alterations to selected genes, enabling exploration of new regions of the solution space and reducing the risk of convergence to local optima.

The optimization procedure was iterated for a specified number of generations or until a satisfactory fitness level was achieved. Throughout these iterations, the GA progressively improved the overall fitness of the population, converging toward an optimal set of parameters. The final outcome was an optimized pair of *λ* and *L* values that minimized the number of out-of-control observations, thereby prioritizing the specificity of the EWMA control chart in detecting process variations and reducing unnecessary alarms, with an acknowledged trade-off in sensitivity.

## 3. Results

This section presents the results of the proposed framework, including descriptive statistics of the dataset, feature selection based on information functions, risk prediction modeling, and the design and genetic algorithm–based optimization of EWMA control charts. Collectively, these results demonstrate the effectiveness of the proposed approach in improving treatment monitoring and supporting data-driven decision-making in thyroid cancer care.

### 3.1. Descriptive statistics of the study dataset

The descriptive statistical analysis of the dataset, presented in Table 2, comprising 80 patients with thyroid cancer revealed that participant ages ranged from 21 to 79 years, with a mean age of 50.74 years (SD = 18.06). Male patients accounted for 54% of the study population. The mean body mass index (BMI) was 26.58 kg/m^2^ (SD = 4.71), indicating that, on average, patients were in the overweight category. Smoking and alcohol consumption were each reported by 54% of participants. Patients presented with an average of 1.93 comorbid conditions (SD = 1.39), and 40% reported a family history of thyroid cancer. Previous thyroid surgery was documented in 46% of cases.

**Table 2:** Descriptive statistics of thyroid cancer patient characteristics (N = 80).

Feature	Description (Unit)/ Categories	Mean	SD	Min	Max	Median	Mode
Age	Age (years)	50.74	18.06	21	79	48.5	63
Gender	0 = Female, 1 = Male	0.54	–	0	1	1	1
BMI	Body Mass Index (kg/m²)	26.58	4.71	18.6	34.8	26.65	29
Smoking_Status	0 = No, 1 = Yes	0.54	–	0	1	1	1
Alcohol_Consumption	0 = No, 1 = Yes	0.54	–	0	1	1	1
Comorbidities	Number of comorbid conditions	1.93	1.39	0	4	2	1
Family_History	Family history of thyroid cancer 0 = No, 1 = Yes	0.4	–	0	1	0	0
Previous_Thyroid_Operation	Previous thyroid surgery 0 = No, 1 = Yes	0.46	–	0	1	0	0
Tumor_Size	Maximum tumor diameter (cm)	2.92	1.17	1.1	5	2.95	1.1
Surgery_Type	0 = Partial, 1 = Total	0.56	–	0	1	1	1
TG_Level_Before_Treatment	Thyroglobulin level before treatment (ng/mL)	5.2	2.97	0.2	10	5.2	1.4
TG_Level_Stage_1	Thyroglobulin level at stage 1 (ng/mL)	4.16	2.64	0.3	9.9	3.7	0.5
TG_Level_Stage2	Thyroglobulin level at stage 2 (ng/mL)	5.28	2.8	0.1	9.8	5.45	1.6
Time_Between_Stages	Time Between Treatment Stages (days)	104.48	43.45	30	179	102	61
Radiation_Therapy	0 = No, 1 = Yes	0.46	–	0	1	0	0
Radioactive_Iodine_Dose	Dose of radioactive iodine administered (mCi)	65.8	19.64	33.6	99.3	65.35	41.5
Outcome	Treatment Outcome 0 = Unsuccessful, 1 = Successful	0.43	–	0	1	0	0

Regarding disease characteristics and treatment, the mean tumor size was 2.92 cm (SD = 1.17). Total thyroidectomy was performed in 56% of patients. The mean time interval between stage 1 and stage 2 treatments was 104.48 days (SD = 43.45). Radiation therapy was received by 46% of the patients. Treatment outcomes indicated that only 43% of patients achieved a successful outcome, with the majority experiencing unsuccessful treatment. These findings underscore the complexity of patient profiles and treatment processes, as well as substantial variability in treatment responses, highlighting the need for more precise and personalized treatment monitoring strategies.

### 3.2. Feature selection using information functions

The Information Functions algorithm was employed to identify the most influential features by estimating the mutual information between each predictor and the outcome variable. The selected features and their corresponding mutual information scores are summarized in Table 3. As indicated in Table 3, thyroglobulin levels before treatment, at stage 1, and at stage 2 exhibited the highest mutual information values and were therefore selected as the primary predictors for subsequent modeling.

**Table 3:** Feature selection results using the information functions algorithm.

Feature	Mutual Information
Gender	0.02489451
BMI	0.01923225
Alcohol_Consumption	0.01679837
Comorbidities	0.08088223
Family_History	0.08011171
Previous_Thyroid_Operation	0.08200295
Surgery_Type	0.07006699
TG_Level_Before_Treatment	0.05372928
TG_Level_Stage_1	0.05428636
TG_Level_Stage_2	0.13228087
Time_Between_Stages	0.07057064
Radiation_Therapy	0.04973888

### 3.3. Risk prediction modeling and performance evaluation

The predictive performance of multiple machine learning models was evaluated to identify the optimal risk prediction model for thyroid cancer patients. Table 4 presents the performance metrics, accuracy, 5-fold cross-validation accuracy, AUC, and F1 score, for all models. Overall, the MLP and DNN models outperformed other approaches across all metrics, with the MLP achieving the highest Cross**-**Validation Accuracy (0.84), AUC (0.88) and F1 score (0.91). These results indicate that more complex models, such as MLP and DNN, are better suited to capturing the nonlinear relationships in the data, thereby providing more accurate risk predictions for thyroid cancer patients.

**Table 4:** Comparative performance of machine learning models for thyroid cancer risk prediction across multiple evaluation metrics.

Model	Accuracy	Cross-Validation Accuracy	AUC	F1 Score
Linear Regression	0.74	0.81	0.76	0.79
Random Forest	0.75	0.79	0.79	0.78
SVM	0.76	0.8	0.82	0.79
KNN	0.72	0.77	0.8	0.83
DNN	**0.78**	0.82	0.84	0.84
MLP	0.77	**0.84**	**0.88**	**0.91**

### 3.4. Results of control chart construction

The previously trained MLP model was applied to generate predicted risk scores for key features related to thyroid cancer treatment. The mean (μ) and standard deviation (σ) of these predicted risk scores were calculated and are summarized in Table 5. These baseline parameters were then used to construct the EWMA control chart, enabling the computation of EWMA statistics and the establishment of corresponding control limits for process monitoring.

**Table 5:** Descriptive statistics of MLP-predicted risk scores for key thyroid cancer treatment features.

Feature	Mean (μ)	Standard Deviation (σ)
TG_Level_Before_Treatment	3.83	2.13
TG_Level_Stage1	7.67	5.68
TG_Level_Stage2	4.81	2.20
Tumor_Size	3.54	0.64
Time_Between_Stages	96.28	3.51
Radioactive_Iodine_Dose	56.76	7.60

Using the estimated baseline parameters, the EWMA statistics, along with the upper control limit (UCL) and lower control limit (LCL), were calculated for each key feature. These EWMA control charts were designed to detect significant deviations in predicted treatment risk across features, facilitating timely identification of potential changes in the thyroid cancer treatment process.

### 3.5. Optimization results of control chart parameters

The initial EWMA control chart parameters were set to a smoothing parameter of λ=0.15 and a control limit multiplier of L=3.5. Using the GA, these parameters were optimized to λ=0.20 and L=4.0, as summarized in Table 6. The increase in λ assigns greater weight to recent observations, enhancing the chart’s responsiveness to emerging changes, while the adjustment of L modifies the control limits to achieve a better balance between sensitivity and false alarm rates.

**Table 6:** The initial and optimized parameter values.

Parameter	Initial Value	Optimized Value
λ (Smoothing parameter)	0.15	0.2
L (Control limits)	3.5	4

Table 7 presents the initial EWMA values and the corresponding initial and optimized control limits for the predicted risk scores. Comparison of control charts before and after optimization, illustrated in Fig 4, revealed that the GA-optimized EWMA charts (green line) exhibit smoother trajectories and fewer out-of-control signals than the original EWMA charts (orange line) across all monitored features. The optimized EWMA curves show reduced variability and, in some intervals, appear as nearly horizontal segments, reflecting convergence of the EWMA statistic under stable predicted risk values. This behavior is a direct consequence of the increased smoothing parameter and indicates improved noise suppression rather than a loss of sensitivity. Overall, these findings demonstrate that GA-optimized parameters reduce false alarms while preserving sensitivity to meaningful process changes. The consistency of this improvement across features suggests that GA-based optimization enhances overall control chart performance rather than feature-specific behavior.

**Table 7:** EWMA statistics and control limits for key thyroid cancer treatment features.

Feature	EWMA	Initial UCL	Initial LCL	Optimized LCL	Optimized UCL
TG_Level_Before_Treatment	3.83	1.71	5.95	1.00	6.67
TG_Level_Stage1	7.67	2.01	13.34	0.09	15.25
TG_Level_Stage2	4.81	2.62	7.01	1.88	7.75
Tumor_Size	3.54	2.89	4.18	2.68	4.40
Time_Between_Stages	96.28	92.78	99.77	91.60	100.95
Radioactive_Iodine_Dose	56.76	49.19	64.33	46.64	66.89

**Fig 4:**
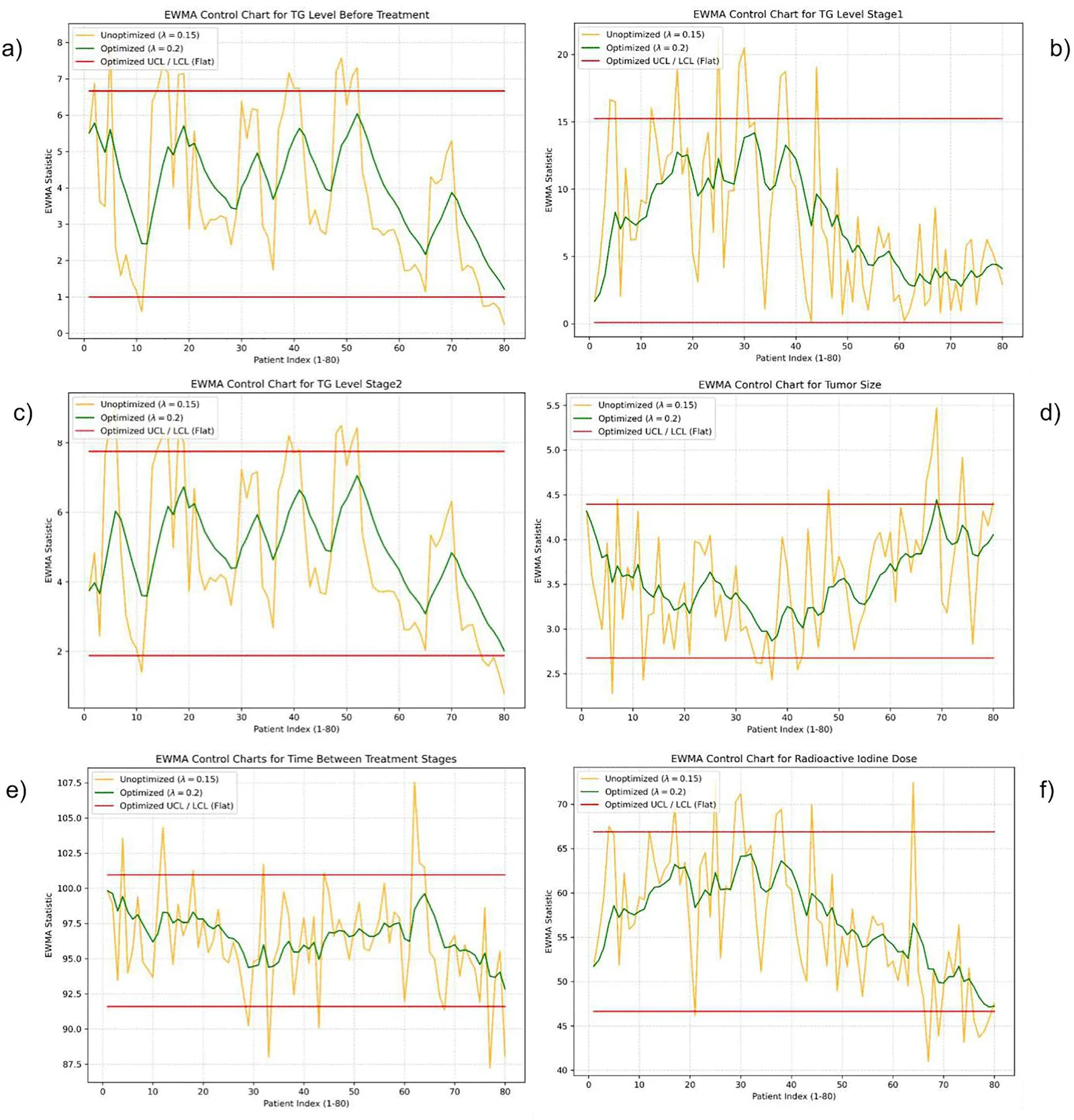
Comparison of EWMA control charts before and after genetic algorithm–based optimization for key thyroid cancer treatment features. Each panel illustrates the performance of the Exponentially Weighted Moving Average (EWMA) control chart for a selected thyroid cancer treatment feature before and after parameter optimization. The orange line represents the EWMA statistic computed using the initial parameter settings (λ = 0.15, L = 3.5), while the green line shows the EWMA statistic after optimization using a genetic algorithm (λ = 0.20, L = 4.0). The red horizontal lines denote the upper and lower control limits (UCL and LCL), which define the thresholds for detecting statistically significant process variations. Panels correspond to: (a) Thyroglobulin (TG) level before treatment; (b) Thyroglobulin level at stage 1; (c) Thyroglobulin level at stage 2; (d) tumor size; (e) time between treatment stages; and (f) radioactive iodine dose.

As an example of the chart’s operational use, in the Tumor Size panel (Fig 4d), the EWMA statistic remains within the control limits for the majority of the monitored sequence, an in-control state consistent with expected variation, before crossing the upper control limit in the later portion of the trajectory. This out-of-control signal indicates a change beyond expected variability and, in clinical use, would flag the corresponding patient for prioritized assessment and follow-up.

## 4. Discussion

This study presents a framework that integrates deep learning–based risk prediction with genetic algorithm-optimized statistical process monitoring to enhance the monitoring of thyroid cancer treatment processes. By jointly leveraging machine learning, information-theoretic feature selection, and optimized EWMA control charts, the proposed approach addresses key limitations of traditional monitoring methods, which often lack sensitivity to subtle yet clinically meaningful changes in patient status.

The results demonstrate that the MLP model outperformed several conventional machine learning models in predicting patient risk, highlighting the importance of nonlinear modeling for capturing the complex relationships inherent in clinical and treatment-related data. The identification of thyroglobulin level at different treatment stages as the most informative predictor aligns with its clinical use as a key biomarker in thyroid cancer management [28,29]. Beyond prediction, a major contribution of this study is the application of EWMA control charts to machine learning-derived risk prediction scores rather than raw clinical measurements, thereby enabling proactive process monitoring by translating predictive insights into actionable monitoring signals. Genetic algorithm–based optimization of EWMA parameters improved chart performance, as evidenced by smoother EWMA trajectories, reduced false alarms, and preserved sensitivity to meaningful process changes across all monitored features. The consistent improvement across features suggests that GA-based optimization enhances overall chart behavior rather than tailoring performance to individual variables. In particular, the higher smoothing parameter (λ) assigns greater weight to recent observations, enhancing the chart’s responsiveness to emerging changes while maintaining stability.

From a clinical and operational perspective, the framework could be integrated into hospital EMR systems as a clinical decision-support tool to enable dynamic, sequential monitoring of treatment processes. At each clinical update, such as new laboratory results, surgical procedures, or follow-up visits, the MLP model will generate updated patient-specific risk estimates, which will be sequentially incorporated into the EWMA monitoring component and will evaluate each patient’s treatment trajectory relative to expected performance over time, enabling continuous longitudinal assessment rather than reliance on isolated cross-sectional evaluations. When the EWMA statistics exceed predefined control limits, the system generates an automated alert notifying the clinical team that the patient may require additional evaluation or follow-up. In practice, this approach enables risk-stratified monitoring, whereby patients with stable or low predicted risk continue routine surveillance, while those demonstrating elevated or worsening risk profiles are prioritized for targeted diagnostic testing or intervention. This potential workflow may improve the allocation of clinical resources, minimize alarm fatigue, and ultimately improve patient outcomes while reducing complications and healthcare costs. Beyond thyroid cancer, the framework may provide a methodological template for monitoring other diseases involving longitudinal treatment data and complex patient trajectories [30] and may support data-driven quality assurance and performance evaluation.

The framework used in this study is related to, but conceptually distinct from, the established family of risk-adjusted control charts used in healthcare surveillance. Risk-adjusted cumulative sum (CUSUM) charts [31,32] and risk-adjusted EWMA charts [33] were developed to monitor clinical outcomes [34], most commonly binary events such as surgical mortality, while accounting for patients’ preoperative risk [14,35]. These methods typically use logistic regression for risk adjustment and are designed to evaluate aggregate performance at the institutional level. In contrast, our approach serves as a longitudinal, patient-level risk surveillance rather than case-mix-adjusted performance monitoring and differs in both its monitoring target and methodology. More recent studies have integrated neural networks with risk-adjusted outcome monitoring, including evolutionary artificial neural network (ANN)-based control charts evaluated using the average run length criterion [36–39]. Our contribution differs from these approaches by focusing on continuous risk-score trajectories rather than binary clinical outcomes. A direct empirical comparison with conventional risk-adjusted CUSUM and EWMA charts would help clarify the relative strengths and limitations of each approach and represents an important direction for future research.

Despite its contributions, this study has several limitations. The dataset was relatively modest in size and derived from a single institution, which may limit the generalizability of the findings. This constraint is inherently linked to the nature of thyroid cancer as a relatively rare malignancy, which presents significant challenges in data acquisition. However, the adequacy of this sample size for monitoring purposes was previously validated through simulation analyses on the same cohort [15]. Also, an independent Phase I calibration cohort was not available, and the in-control parameters were therefore estimated from the same dataset used for Phase II monitoring. This is a recognized limitation, as the use of estimated rather than independently specified in-control parameters can affect EWMA run-length performance [23]. While limited training data frequently hinders the development of predictive models, a key contribution of this work is the achievement of high predictive accuracy and efficiency despite these constraints. This demonstrates the framework’s robustness in rare disease contexts where large-scale datasets are unavailable.

The monitored risk score is derived from short per-patient trajectories with an unknown distribution. Under these conditions, reliable estimation of average run length (ARL) would require assumptions that are not sufficiently supported by the available data. A formal ARL evaluation using an independent calibration cohort and a larger, multi-institutional dataset therefore remains an important area for future investigation. Additionally, the genetic algorithm objective function used in this study minimizes the frequency of out-of-control signals and therefore emphasizes specificity. As it does not distinguish between true and false alarms, it does not simultaneously optimize sensitivity. A joint sensitivity–specificity formulation based on average run length could address this limitation by constraining the in-control ARL (ARL₀) to control the false-alarm rate while minimizing the out-of-control ARL (ARL_1_) for clinically meaningful shifts. Given the relatively low-dimensional search space, alternative metaheuristic approaches such as particle swarm optimization or grey wolf optimization may yield comparable or potentially improved results [40]. Therefore, future research should compare these optimization strategies and evaluate adaptive or personalized control limits, real-time data processing, and advanced machine learning architectures using larger, multicenter datasets. Extending this approach to other cancer types and chronic disease management scenarios represents a promising direction for future research.

### Disclosure of generative AI use

ChatGPT (GPT-5.5) was used solely to enhance grammatical accuracy. It was not involved in ideation, study design, data analysis, programming, or any other research-related activities.

## Abbreviations

AUC: Area Under the Curve
ARL: Average Run Length
ANN: Artificial Neural Network
BMI: Body Mass Index
CUSUM: Cumulative Sum
DNN: Deep Neural Network
EMR: Electronic Medical Record
EWMA: Exponentially Weighted Moving Average
GA: Genetic Algorithm
KNN: K-Nearest Neighbors
LCL: Lower Control Limit
MLP: Multilayer Perceptron
SPC: Statistical Process Control
SVM: Support Vector Machine
UCL: Upper Control Limit
